# A Temperature-Hardened Sensor Interface with a 12-Bit Digital Output Using a Novel Pulse Width Modulation Technique

**DOI:** 10.3390/s18041107

**Published:** 2018-04-05

**Authors:** Emna Chabchoub, Franck Badets, Frédérick Mailly, Pascal Nouet, Mohamed Masmoudi

**Affiliations:** 1 Grenoble Alpes University, CEA, LETI, F-38000 Grenoble, France; Franck.Badets@cea.fr; 2 Laboratory of Informatics, Robotics and Microelectronics of Montpellier, University of Montpellier, CNRS, 34000 Montpellier, France; Frederick.Mailly@lirmm.fr (F.M.); nouet@lirmm.fr (P.N.); 3 METS Research Group, National Engineers School of Sfax, Sfax University, Sfax 3029, Tunisia; mohamed.masmoudi@enis.tn

**Keywords:** high temperature, sensor interface, time-domain, injection locked oscillators

## Abstract

A fully integrated sensor interface for a wide operational temperature range is presented. It translates the sensor signal into a pulse width modulated (PWM) signal that is then converted into a 12-bit digital output. The sensor interface is based on a pair of injection locked oscillators used to implement a differential time-domain architecture with low sensitivity to temperature variations. A prototype has been fabricated using a 180 nm partially depleted silicon-on-insulator (SOI) technology. Experimental results demonstrate a thermal stability as low as 65 ppm/°C over a large temperature range from −20 °C up to 220 °C.

## 1. Introduction

An increasing demand for electronic systems working at temperature up and even beyond 200 °C has appeared over the last years for critical applications such as oil drilling, automotive and/or aeronautic uses [1,2,3]. In such applications, it is highly desirable to place the sensor electronic interface as close as possible to the transducer for the sake of signal integrity [4]. Hence, the sensor electronic interface must be able to work in a harsh environment. Nevertheless, while several techniques already exist to improve the robustness of digital circuits against high temperature environments, analog functions are still a bottleneck towards the realization of high temperature system-on-a-chip (SOC). Indeed, analog blocks’ performances degrade with temperature because of the exponential increase of leakage currents and the decrease of both transistors threshold voltage and carrier mobility, that dramatically vary biasing points and lower output impedances and transistors’ intrinsic gain [5]. 

Considering the constraint of thermally stable performance, time-domain sensor interfaces have appeared that are more competitive than classical analog sensor interfaces [6,7,8,9]. The time-domain approach is based on converting the sensor analog output into a time domain signal; a frequency, or a phase shift (for example a pulse width of pulse width modulated (PWM) signal), which can be easily digitized with the use of a time to digital converter (TDC) [10,11]. This approach eliminates the need of low noise amplifiers (LNAs) and analog to digital converters (ADCs) that are both known to be highly sensitive to temperature variations. In addition to that, time domain circuits are quasi-digital circuits that benefit from a higher noise margin and lead to relatively simple architectures with gains in terms of cost, size and power consumption [12,13].

Frequency-based time-domain architectures are based on voltage controlled oscillators (VCOs). They usually suffer from the high non-linearity of the oscillator frequency with respect to the tuning voltage [14]. Recently, Nebhen et al. proposed a solution to improve the linearity of VCOs, but their non-linearity is still as high as 2% [15]. In addition, its frequency instability is higher than 133.3 ppm/°C which leads to a too high overall thermal variation of the sensor interface.

Originally, PWM time-domain sensor interfaces were introduced to generate quasi-digital output signals which are less sensitive to noise coupling when transmitted compared to analog signals. Usually, PWM readout circuits are based on a periodic ramp generation whose output is compared to the analog quantity to be converted. The duration of the generated PWM equals the time difference between the ramp beginning and the comparator decision time, which is in turn proportional to the voltage to be converted. However, thermal stability of PWM-based readout circuits is affected by the thermal stability of the offset voltage of the comparator and of the ramp slope. What’s more, in typical PWM techniques, the degradation of the non-linearity of the ramp slope over the operation temperature range degrades the overall performance of the so-obtained PWM signal. De Smedt et al. in [6], proposed a new PWM technique that uses a ratio-metric measure. Two delay stages, driven by a differential sensor, convert the sensor value into a PWM signal whose duty cycle is the ratio of two delay measurements that are frequency-independent. A 79 ppm/°C thermal drift of the sensor interface has been measured. Glaser et al. in [7], amplified the sensor output voltage by means of an analog amplifier before converting it into a PWM signal. Besides, a switching scheme is used to realize different measurements such as offset and temperature of the die. The performances of the proposed solution are limited by the thermal drift of both offset and gain of the amplifier. Moreover, the increase of the leakage current with temperature degrades the performances of the switching scheme. A two-point calibration is necessary to compensate the temperature offset and to reach a measured ±43 ppm/°C thermal drift.

In this paper, a new PWM-based sensor interface architecture that is able to operate over a wide temperature range is presented. It is based on the use of two injection locked relaxation oscillators to convert the transducer analog output into a PWM signal whose pulse width is defined by the difference of phase between both oscillators. Contrary to previous published works, where the PWM signal is transmitted into an ambient temperature environment for performing the signal processing [7], the signal processing of the obtained PWM signal is performed in situ, in the high temperature environment, by means of a TDC. The main feature of the presented architecture is its robustness to the thermal variations of its constitutive blocks. The proposed architecture exhibits a very low thermal sensitivity of 65 ppm/°C which is obtained without the need of any calibration.

The paper is organised as follows: Section 2 presents the principle of the sensor interface based on a PWM technique using injection locked oscillators (ILOs). Section 3 describes the architecture of the proposed high temperature sensor interface and its detailed implementation. Experimental and simulated results over a wide temperature range are reported in Section 4 where characterization results of the fabricated prototype are compared to results obtained by simulation. Finally, Section 5 concludes the presented work.

## 2. Sensor Interface Principle Based on Voltage to Pulse Width Modulated signal Conversion

The proposed voltage to PWM conversion is based on ILOs used as phase shifters. The so-obtained pulse width is proportional to the phase shift of the ILO with respect to a reference signal. This section briefly recalls the ILO-based voltage to PWM conversion which is behind the principle of the sensor interface architecture.

### 2.1. Injection Locked Oscillators

**Figure 1:**
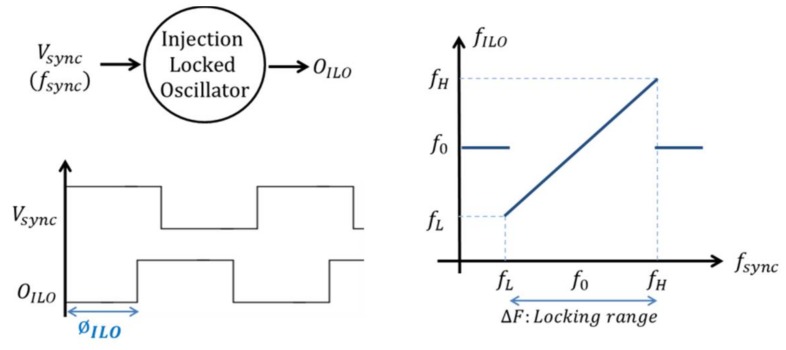
Property of Injection Locked Oscillators (ILOs).

It is well known that an oscillator can be locked on a parasitic signal if its frequency is close to the oscillator free running frequency [16] (Figure 1). ILOs are particular circuits that take advantage of this property. They consist in oscillators with an input on which a varying signal is applied. ILOs are commonly used in radio frequency (RF) circuits as frequency multipliers or frequency dividers. In Huntoon et al. [17], Huntoon and Weiss proposed a theoretical study of ILOs in the case of a small amplitude harmonic locking signal. In this case, they showed that the injected signal can be modelled as a small variation of the impedance seen at the point where the locking signal is applied. With this assumption, it was shown that the tracking process of the ILO is given by:

(1) $$\frac{1}{2\pi }\frac{\partial \phi_{ILO}}{\partial t}=(f_{sync}-f_{0})-\frac{\left|V_{S0}\right|\left|E_{F}\right|}{\left|I_{0}\right|}\cos(\phi_{ILO}+\beta )$$

where *Φ_ILO_* is the ILO phase shift (i.e., the phase difference between the ILO output *O_ILO_* and the locking signal), *V_S_*_0_ is the voltage magnitude of the locking signal and *I*_0_ the output current magnitude. *E_F_* is a called compliance factor (|*E_F_*| is its amplitude and *β* is its phase) which is the ratio of the variation of the oscillator frequency with respect to the impedance variation. *f_sync_* is the locking signal frequency and *f*_0_ is the oscillator free running frequency. 

Once locked, the ILO output tracks the locking signal phase variation and Equation (1) could be rewritten as:

(2) $$\left(f_{sync}-f_{0})=\frac{\left|V_{S0}\right|\left|E_{F}\right|}{\left|I_{0}\right|}\cos(\phi_{ILO}+\beta \right)$$

Noting that cosinus function is lower than 1 and greater than −1, the locking range is easily obtained as:

(3) $$\Delta F=2\frac{\left|V_{S0}\right|\left|E_{F}\right|}{\left|I_{0}\right|}$$

Thus, according to Huntoon and Weiss, the ILO locking range depends on the amplitude of the locking signal *V_S_*_0_, the oscillator output current magnitude *I*_0_ and the compliance factor *E_F_* which depends only on the oscillator topology [16].

From Equation (2), the ILO phase shift, once the ILO is locked, is extracted:

(4) $$\phi_{ILO}=\mathrm{acos}(\frac{2(f_{sync}-f_{0})}{\Delta F})$$

Therefore, once locked, the ILO phase shift only depends on the difference between the locking and the oscillator free running frequencies and on the locking range. 

This property is very interesting as it allows the use of ILOs as phase shifters by keeping the locking frequency fixed and tuning the ILO free running frequency with an analog command as it is done for voltage or current controlled oscillators [18,19]. Thus, ILOs are used as analog to time converters in time domain sensor interfaces.

### 2.2. Pulse Width Modulation-Based Sensor Interface Using Injection Locked Oscillator

The proposed PWM circuit is based on the previously mentioned properties of ILOs. The sensor voltage *V_S_* is used to control the free running frequency of the ILO *f*_0_. Then, if a fixed locking frequency is used, the obtained phase shift *Φ_ILO_* is a function of the free running frequency and thus, the obtained phase shift is a function of the input voltage *V_S_*.

**Figure 2:**
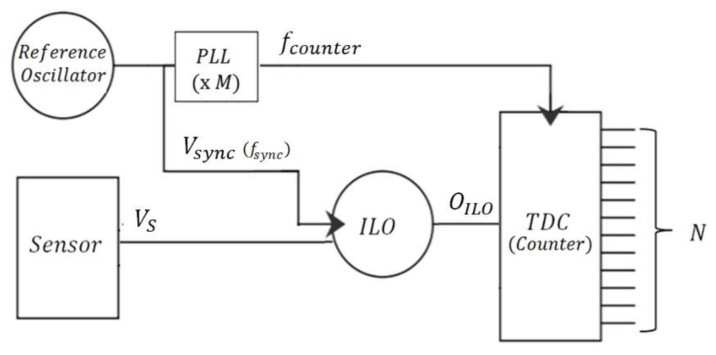
ILO-based Pulse Width Modulation (PWM) sensor interface.

Figure 2 depicts the principle of an ILO-based PWM sensor interface. It is composed of an ILO-based phase shifter and a counter-based TDC. This counter is clocked by a counting frequency that is a multiple of the locking frequency. The locking signal is generated from an oscillator with the same topology as the ILO.

**Figure 3:**
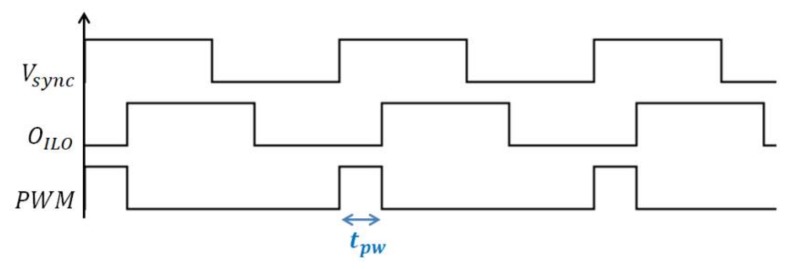
Representation of the output PWM signal.

The time shift *t_pw_* between the locking signal *V_sync_* and the ILO output *O_ILO_* is linked to the ILO phase shift by Equation (5) and it is hence a function of *V_S_* (Figure 3):

(5) $$t_{pw}=\frac{\phi_{\mathrm{ILO}}}{2\pi }T_{sync}$$

where *T_sync_* is the period of the locking signal.

Since the counter resolution (i.e., the counting period *T_counter_*) is *T_sync_*/*M* (*M* is the PLL (Phase Locked Loop) frequency multiplication factor), the TDC output is given by:

(6) $$N=\frac{t_{pw}}{T_{counter}}=M\frac{t_{pw}}{T_{sync}}$$

Based on Equations (5) and (6) is written as:

(7) $$N=\frac{M}{2\pi }\phi_{ILO}$$

Based on Equations (3) and (4) from Huntoon and Weiss theory, the digital output of the sensor interface is expressed as:

(8) $$N=\frac{M}{2\pi }\mathrm{acos}((f_{sync}-f_{0})\frac{\left|V_{S0}\right|\left|E_{F}\right|}{\left|I_{0}\right|})$$

The locking signal is generated from an oscillator having identical topology as that of the ILO free running oscillator, thus, the locking frequency *f_sync_* has the same temperature dependency as the ILO free running oscillator *f*_0_. Hence, their difference (*f_sync_* − *f*_0_) will be insensitive to temperature variations. What is more, the ratio between |*V_S_*_0_| and |*I*_0_| (i.e., |*V_S_*_0_|/|*I*_0_|) can be made insensitive to temperature variations if they have both the same temperature coefficient. This can be achieved if, for example, *V_S_*_0_ is derived from the same biasing circuit as that of the oscillator. Moreover, the frequency multiplication factor M is temperature independent. In fact, the frequency multiplier (i.e., the PLL) is designed to be always locked whatever temperature is; thus, it realizes the same frequency multiplication factor all over the operation temperature range. In addition to that, the compliance factor *E_F_* depends on the ILO passive components (capacitances for example) which have a low temperature coefficient; hence *E_F_* has a low thermal variation. As a consequence, a low temperature dependence could be expected for the digital output of such sensor interface architecture.

This architecture principle is adapted to any kind of ILOs and it leads to low temperature sensitivity. Nevertheless, in the case of harmonic locking signal, the overall architecture is limited by the non-linearity of the acos function that links the digital output to the frequency difference between the locking frequency *f_sync_* and the ILO free running frequency *f*_0_ (Equation (8)). Indeed, the linear behaviour of the acos function is limited to a phase shift range of π/2. Therefore, the linear range of the harmonic ILO-based PWM sensor interface will be limited to half of its dynamic range (the full dynamic range corresponds to a phase shift range of π).

In this paper, we introduce an ILO having an extended linear range which is based on a relaxation oscillator and a non-harmonic injection scheme [20]. An ILO with an extended linear range offers the advantage of a good linear behaviour for the ILO-based PWM sensor interface.

## 3. Architecture of the Proposed High Temperature Sensor Interface

### 3.1. Injection Locked Relaxation Oscillator (ILRO)

**Figure 4:**
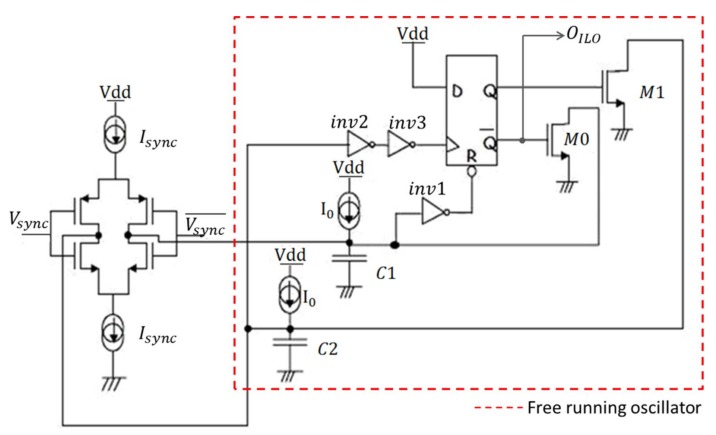
Schematic of the Injection Relaxation Locked Oscillator.

The chosen ILO is based on a relaxation oscillator as depicted in Figure 4 [19,20].

Each ILO is composed of a free running oscillator and a sub-circuit for locking this oscillator with the locking signal as depicted in Figure 4. The free running oscillator works as follows: assuming that at the initial state the output of the oscillator O*_ILO_* is equal to zero, meaning that the D-flip-flop (DFF) output *Q* is at logic “1”, *M*1 is on while *M*0 is off. *C*2 is shorted to ground through *M*1 while *C*1 is charged by a bias current *I*_0_. When the voltage across *C*1 reaches the threshold voltage of the inverter inv1 *V_th_*, the DFF is reset (*Q* is equal to a “0” logic level) and *O_ILO_* is equal to the supply voltage Vdd. Hence, *M*1 is turned off and *M*0 is turned on, then *C*1 is rapidly discharged while the capacitor *C*2 is charged by the same bias current *I*_0_ until the voltage across *C*2 reaches the threshold voltage of the inverter inv2 *V_th_*. Consequently, a rising edge appears on the clock input of the flip-flop and it switches the DFF output to Vdd and *O_ILO_* to zero. The same cycle is then repeated and the frequency of oscillation depends on *I*_0_, *V_th_*, *C*1 and *C*2. 

Inverters inv1 and inv2 are used to ensure a square waveform signal at the DFF clock and reset inputs. The output of the ILO achieves a 50% duty cycle by adopting the same value *C* for *C*1 and *C*2. The free running oscillation frequency is expressed as follows:

(9) $$f_{0}=\frac{I_{0}}{2CV_{th}}$$

where *C* = *C*1 = *C*2. A change in the value of *I*_0_ can easily and linearly modify the free running oscillation frequency.

**Figure 5:**
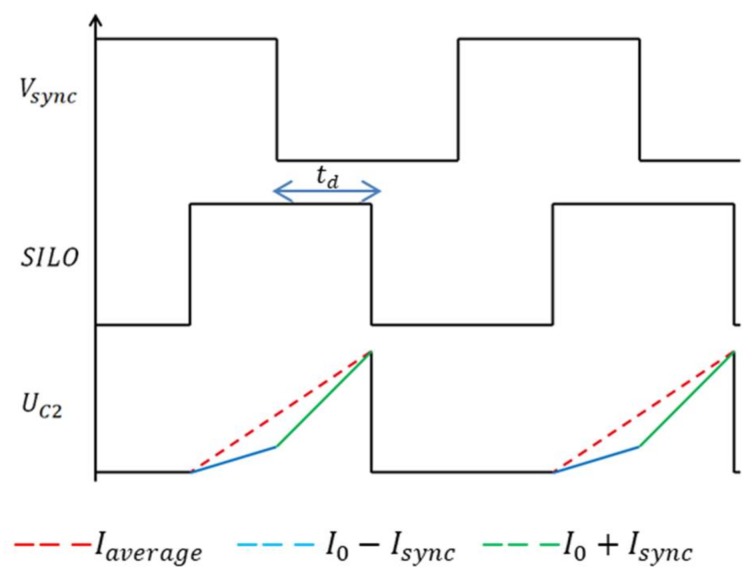
Locking waveforms.

The locking process is performed by periodically modifying the charging current of the capacitors at the locking frequency rate, in such a way that the average charging current on a locking period equals the current which makes the oscillator run at the locking frequency *f_sync_*. The locking process uses a synchronization current *I_sync_* that is either pulled from or pushed into the capacitors of the ILRO. This current is subtracted or added to the bias current *I*_0_ according to the value of the locking signal *V_sync_*. For example, in addition to *I*_0_, a current −*I_sync_* flows into *C*2 at high level of *V_sync_*, while at low level of *V_sync_*, a current +*I_sync_* is added to the charging current of *C*2. Knowing that *C*2 can be charged only when the ILO output is at high level, a delay is created between *V_sync_* and *O_ILO_* as described in Figure 5.

The average charging current of the capacitors is a function of this delay *t_d_*, *I_sync_* and *I*_0_ as expressed in:

(10) Iaverage=(I0+Isync)td+(I0−Isync)(Tsync2−td)Tsync2

As explained before, the so-obtained delay is adjusted in order to make the average charging current of the capacitors *I_average_* equal to the current necessary to get an oscillation frequency which is the same as the locking frequency (*f_sync_*):

(11) $$I_{average}=2f_{sync}CV_{th}$$

Using Equations (9)–(11), the ILO phase shift *Φ_ILO_*, representing the delay between *V_sync_* and *O_ILO_*, can be expressed as:

(12) $$\phi_{ILO}=\frac{\pi }{2}+\frac{\pi CV_{th}}{I_{sync}}(f_{sync}-f_{0})$$

Equation (12) shows that a perfect linear relationship between the ILO phase shift and the frequency difference between the locking frequency and the ILRO free running frequency (*f_sync_* − *f*_0_) is obtained, which is a clear benefit compared to harmonic ILOs.

**Figure 6:**
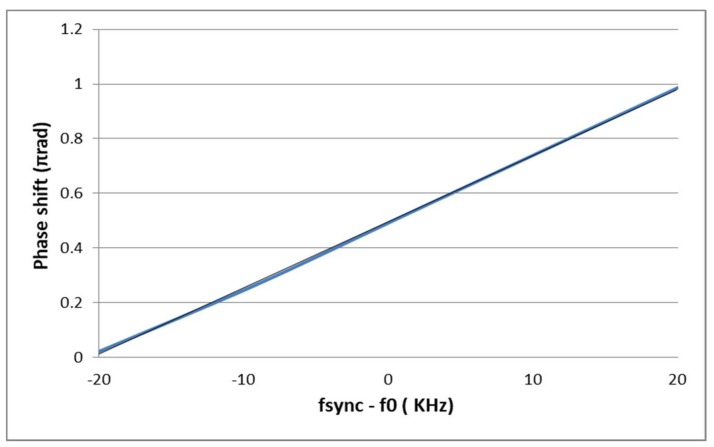
Simulation of the phase shift of the Injection Locked Relaxation Oscillator vs (*f_sync_* − *f_0_*).

Figure 6 depicts the phase shift obtained by simulation of the ILRO of Figure 4, which has been implemented in a 180 nm Partially-depleted Silicon-on-Insulator (PD SOI) process, using ideal frequency source for *f_sync_* and ideal current sources for *I_sync_* and *I*_0_. It shows that the phase shift of the ILRO is linear with respect to (*f_sync_* − *f*_0_) which is in accordance with the theory. A goodness-of-fit of linear regression (*R*^2^) of 0.999 is obtained.

Combining Equations (9) and (12), the phase shift of the ILRO is a linear function of the ILO bias current *I*_0_:

(13) $$\phi_{ILO}=\frac{\pi }{2}+\frac{\pi CV_{th}}{I_{sync}}f_{sync}-\frac{\pi }{2I_{sync}}I_{0}$$

Assuming that the current *I*_0_ is linear with respect to the sensor output voltage, the relation between the ILRO phase shift and the sensor output is linear.

### 3.2. High Temperature Sensor Interface Architecture

**Figure 7:**
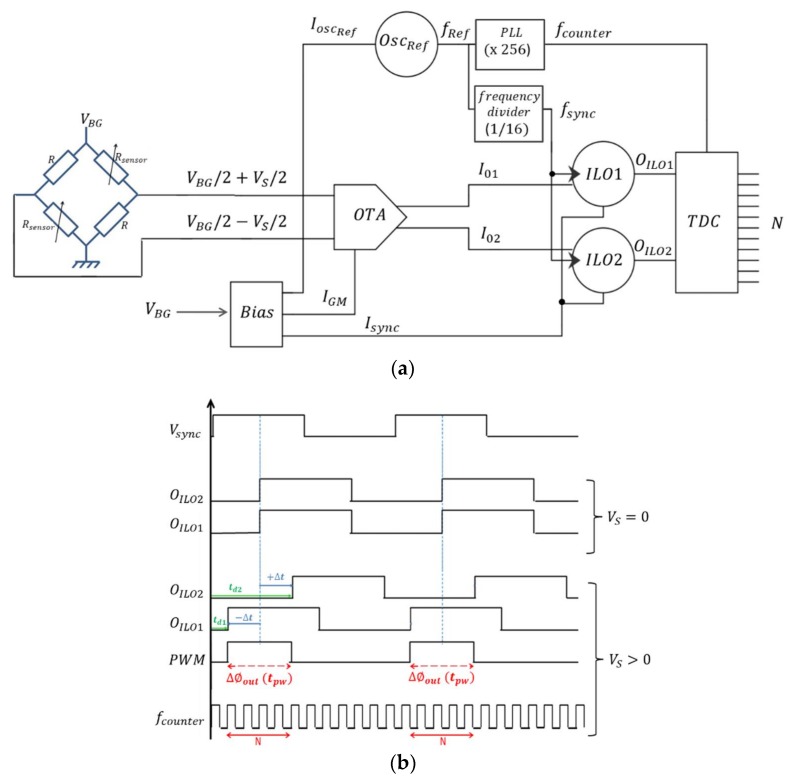
The sensor interface: (**a**) Architecture of the sensor interface; (**b**) Timing diagram (Case of *V_S_* > 0).

The proposed sensor interface, depicted in Figure 7, is dedicated to resistive transducers embedded in a Wheatstone bridge. It converts the sensor differential output voltage into a phase shift difference, using a pair of ILOs, which is then converted into a digital output. 

The differential architecture adopted for this interface has the advantage of rejecting the effects of temperature, process variations and common mode noise. As the output of the Wheatstone bridge is a differential voltage, a transconductance amplifier (OTA) is used to convert the sensor output voltage into a differential current *I*_01_ − *I*_02_. The OTA output currents *I*_01_ and *I*_02_ are then used to control the free running frequencies of ILO1 and ILO2 respectively and thus to control their phase shifts (i.e., *Φ_ILO_*_1_ and *Φ_ILO_*_2_ respectively). 

When the sensor output voltage *V_S_* is equal to zero, both output currents of the OTA are the same and both ILOs have the same free running oscillation frequency, and hence the same phase shift (i.e., *Φ_ILO_*_1_ = *Φ_ILO_*_2_). Then, when *V_S_* is different from zero, the output currents of the OTA are fed into the ILOs, thus, obtaining a simultaneous and opposite variation of their free running oscillation frequencies f_01_ and f_02_ (f_01_ and f_02_ are respectively the free running frequency of ILO1 and ILO2) while their locking frequency is kept constant. This makes their phase shifts, *Φ_ILO_*_1_ and *Φ_ILO_*_2_, vary symmetrically as a function of the sensor output voltage *V_S_*. The difference between the two ILOs phase shifts, i.e., ∆*Φ*_out_ = *Φ_ILO_*_2_ − *Φ_ILO_*_1_, defines the time-domain output of the sensor interface.

Connecting the two ILOs outputs to a simple Exclusive-OR logic gate that is active only when the locking signal *V_sync_* is up, a PWM signal is obtained whose pulse width is defined by ∆*Φ*_out_. 

The obtained PWM signal is then quantized by means of a simple digital counter in order to obtain a digital output value of the sensor output voltage [11,21]. The higher the counter frequency *f_counter_* is, the higher the resolution of the digital output is. Equation (14) expresses the resolution of the sensor interface:

(14) 2Resolution=Tsync/2Tcounter=12fcounterfsync

A high frequency reference oscillator, implemented on the same chip, is used to generate both the locking signal *V_sync_* and the counter clock *f_counter_*.

The counter frequency is generated from this reference oscillator by means of a PLL that realizes a multiplication factor of 256. The same reference oscillator is used to generate the locking signal by means of a frequency divider having a division factor of 16. This choice has been adopted in order to obtain a 11-bit resolution.

The choice of using an intermediate reference oscillator has been adopted as an alternative to the use of a reference oscillator running at *f_sync_* and a PLL realizing a multiplication factor of 4096 that generates the counter clock. In fact, it was decided to lower the PLL multiplication factor to 256 for the sake of its bandwidth. The intermediate reference oscillator runs at *f_counter_*/256. That’s why, the frequency divider must realize a division factor of 16 so that the ratio *f_counter_*/*f_sync_* equals 4096.

Since the sensor interface has the ability to interface sensors with differential output voltages (typically an output of a Wheatstone bridge), an additional bit is used to detect the polarity of the sensor output voltage *V_S_* by detecting which ILO output is in phase advance. For example, when *V_S_* is positive, the output of the first ILO1 is in phase advance with respect to ILO2 output (Figure 7b).

### 3.3. Block Implementation

#### 3.3.1. Biasing Block

**Figure 8:**
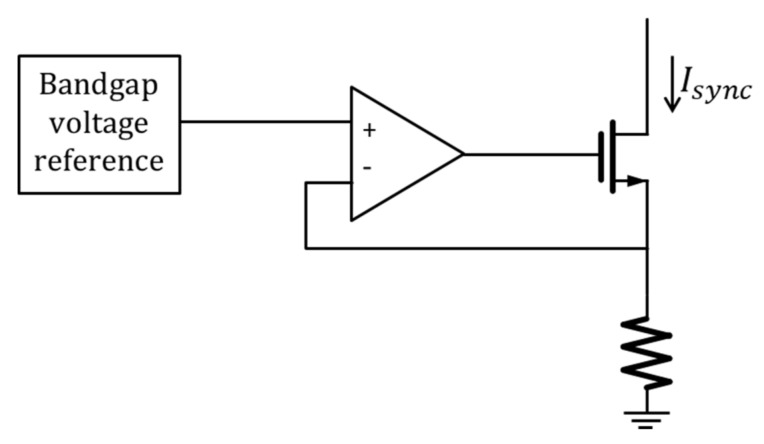
Bandgap-based current reference.

The biasing block generates the synchronisation current of the ILO *I_sync_*. It is based on a bandgap voltage reference as shown in Figure 8. The synchronisation current is expressed as:

(15) $$I_{sync}=\frac{V_{BG}}{R_{Bias}}$$

where *R_Bias_* is the bias resistance of the biasing block and *V_BG_* is the bandgap voltage reference.

The current *I_GM_*, which is the bias current of the OTA, is generated from the synchronisation current by means of a current mirror.

#### 3.3.2. Transconductance Amplifier (OTA)

**Figure 9:**
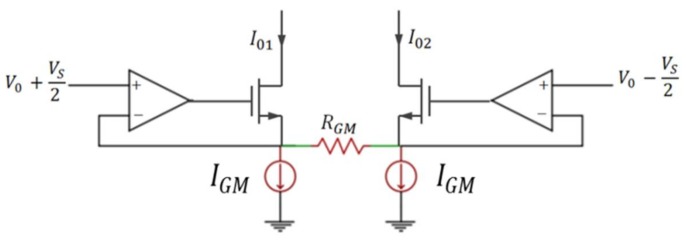
Degenerated transconductance amplifier (OTA) with local feedback.

The transconductance amplifier (OTA) converts the sensor output voltage *V_S_* into two currents with symmetrical variations, *I*_01_ and *I*_02_, which are the bias currents of ILO1 and ILO2 respectively. As the first block encountered in the sensor interface, the linearity of the OTA is an essential specification so that the sensor interface achieves a good linearity. A degenerated OTA with local feedback is adopted to reach the linearity requirement [22,23]. Figure 9 illustrates the implementation of the transconductance amplifier. The local feedback is performed via two side amplifiers which must have a high and thermally stable gain so that the OTA linearity does not degrade with temperature. The constant-gm biasing technique has been used for this purpose [24].

Considering that the two side amplifiers are identical, the difference between the OTA output currents is then given by:

(16) $$I_{01}-I_{02}=\frac{2}{R_{GM}}V_{S}$$

where *R_GM_* is the resistance of the OTA (Figure 9).

**Figure 10:**
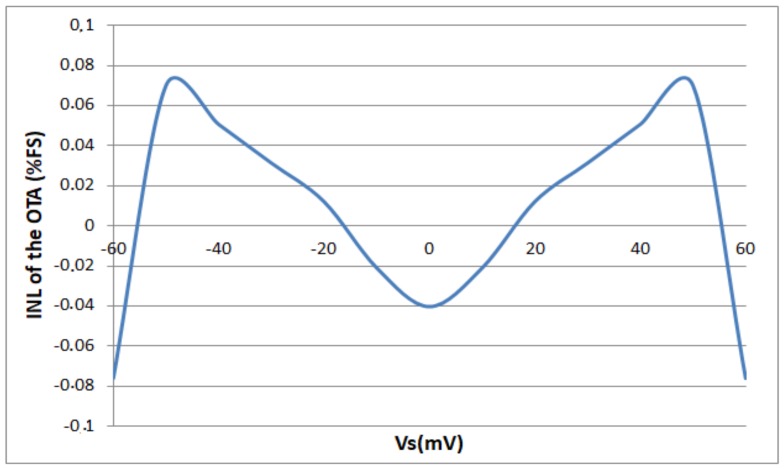
Simulation results of the integral non-linearity of the degenerated OTA with local feedback.

Figure 10 presents the simulated integral non-linearity (INL) error of the proposed OTA. It is lower than 0.08% of the full scale for an input voltage range equal to ±60 mV which is a range consistent with a large number of sensors.

### 3.4. Robustness of the Architecture against Temperature

The pulse width *t_pw_* of the output PWM signal is equivalent to *Φ_ILO_*_2_ − *Φ_ILO_*_1_ (Figure 7b) as expressed in the following equation:

(17) $$t_{pw}=\frac{\phi_{ILO2}-\phi_{ILO2}}{2\pi f_{sync}}=\frac{\Delta \phi_{out}}{2\pi f_{sync}}$$

Therefore, the digital output of the sensor interface is defined as:

(18) $$N=\frac{t_{pw}}{T_{counter}}=\frac{\phi_{ILO2}-\phi_{ILO2}}{2\pi }\frac{f_{counter}}{f_{sync}}$$

where *T_counter_* is the counter period.

Based on Equations (13) and (16), the difference between the two ILOs phase shifts, *Φ_ILO_*_2_ − *Φ_ILO_*_1_, is expressed as:

(19) $$\phi_{ILO2}-\phi_{ILO2}=\frac{\pi }{2I_{sync}}(I_{01}-I_{02})=\frac{\pi }{R_{GM}I_{sync}}V_{S}$$

Using Equations (15) and (19) can be written as:

(20) $$\phi_{ILO2}-\phi_{ILO2}=\pi \frac{R_{Bias}}{V_{BG}R_{GM}}V_{S}$$

Then the digital output of the sensor interface is:

(21) $$N=\frac{1}{2}\frac{R_{Bias}}{R_{GM}}\frac{f_{counter}}{f_{sync}}\frac{V_{S}}{V_{BG}}$$

**Figure 11:**
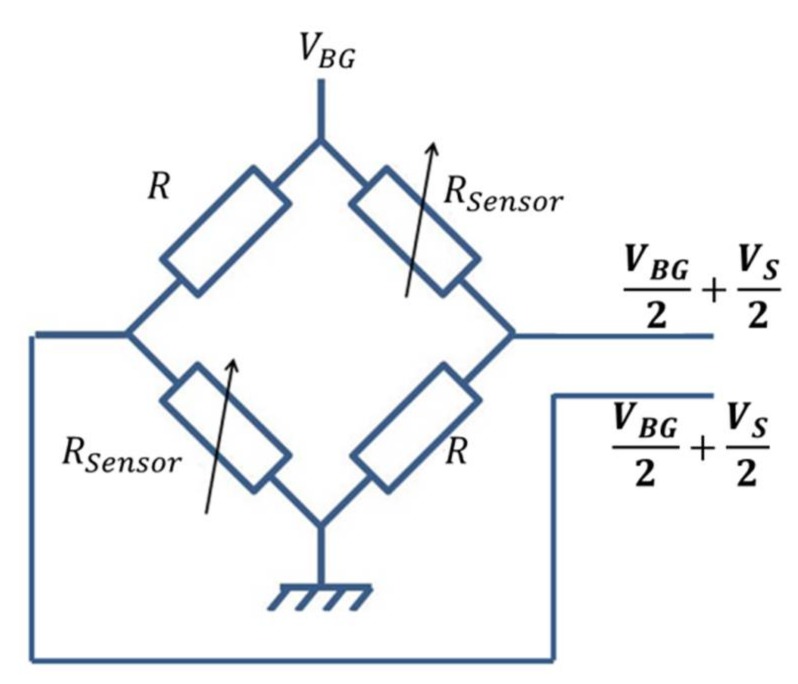
Wheatstone bridge sensor.

In this interface, the Wheatstone bridge is biased by the bandgap voltage reference used for the biasing current generation as depicted in Figure 11.

In this condition, the sensor output voltage *V_S_* is expressed as:

(22) $$Vs=\frac{\Delta R_{sensor}}{2R+\Delta R_{sensor}}V_{BG}$$

where:

$$\Delta R_{sensor}=R_{sensor}-R$$

Consequently, by combining Equations (21) and (22), the contribution of *V_BG_* is eliminated. Hence, the sensor interface output depends only on a ratio of two resistances and a ratio of two frequencies as written below:

(23) $$N=\frac{1}{2}\frac{R_{Bias}}{R_{GM}}\frac{f_{counter}}{f_{sync}}\frac{\Delta R_{sensor}}{2R+\Delta R_{sensor}}$$

Therefore, a low temperature dependency is achieved. Indeed, the ratio *f_counter_*/*f_sync_* is temperature independent because the PLL and frequency divider use the same reference frequency. Moreover, all the resistors embedded in the resistive Wheatstone bridge are of the same nature so that the ratio of resistances is also independent of the temperature variations. Furthermore, using the same kind of resistance for *R_Bias_* and *R_GM_*, their temperature coefficient is the same and their ratio (i.e., *R_Bias_*/*R_GM_*) is then insensitive to temperature variations. For these reasons, the thermal dependency of the digital output should be low.

## 4. Simulations and Experimental Results

The sensor interface is initially designed to interface sensors with an output voltage having a dynamic range of ±60 mV. In fact, the ILO phase shifter has been designed so that the 60 mV input range corresponds to the maximum π phase shift. However, the simulations and experimental results presented in this paper are limited to a ±40 mV input dynamic range because better performances are obtained on this input range compared to the performances obtained on the overall ±60 mV input dynamic range (the performances degrade in terms of linearity and thermal stability).

### 4.1. Simulation Results

The sensor interface is simulated as a function of an input voltage having a ±40 mV full scale which emulates the sensor differential output voltage *V_S_*.

#### 4.1.1. Characteristic Function: Thermal Stability and Linearity

**Figure 12:**
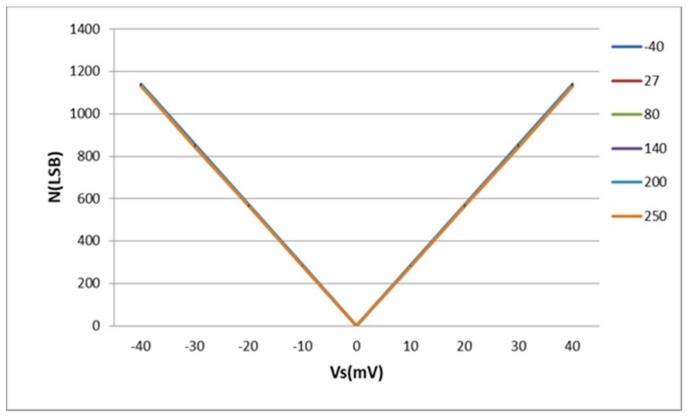
Simulated characteristic function of the sensor interface [−40 °C, 250 °C].

The characteristic function the sensor interface (*N* as a function of *V_S_*) is represented in Figure 12 over an operation temperature range from −40 °C to 250 °C.

**Figure 13:**
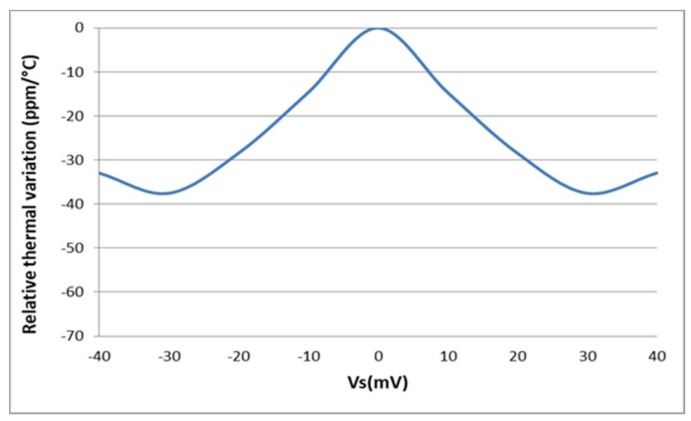
Simulated relative thermal variation of the sensor interface.

Figure 12 shows that the circuit gain is about 30 LSB/mV whatever temperature is. The relative thermal variation, defined in the Equation (24), is used to evaluate the temperature dependence of the sensor interface and plotted in Figure 13:

(24) $$\Delta N(ppm/{}^{\circ}C)=\frac{\Delta N_{absolute}}{N_{FS}\Delta T}10^{6}$$

where:

(25) $$\Delta N_{absolute}=N(T_{\max})-N(T_{\min})$$

where *T_min_* and *T_max_* are respectively the lower and the upper limit of the operation temperature range *∆T* and *N_FS_* is the output full scale.

A negative thermal variation is obtained because the digital output *N* is a decreasing function of the temperature. The absolute value of this thermal variation is lower than 38 ppm/°C over a ±40 mV input dynamic range. 

A zero thermal variation is obtained at zero *V_S_* because the sensor interface is perfectly symmetrical (i.e., *Φ_ILO_*_2_ − *Φ_ILO_*_1_ is always zero at zero *V_S_* over the entire operation range).

**Figure 14:**
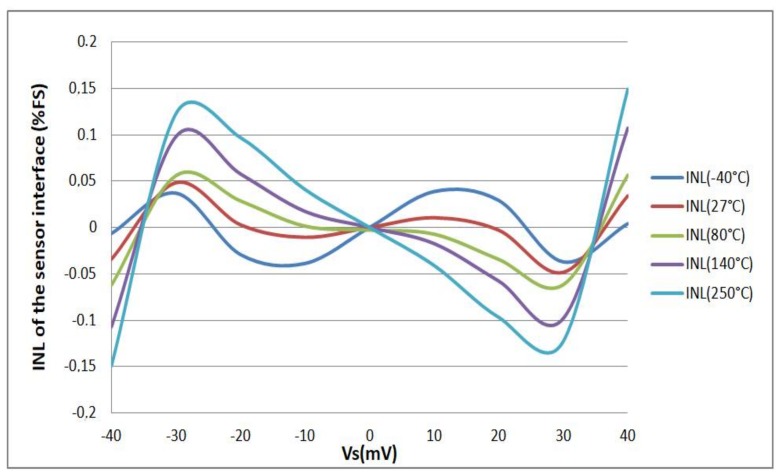
Simulated Integral Non-Linearity (INL) over a ±40 mV input dynamic range.

Simulation results show also the good linearity of the sensor interface. The simulated INL, which is defined as the ratio of the difference between the simulated value and the linear fitted value over the linear fitted value, is lower than 0.15% over the wide operation temperature range (Figure 14). This good linearity is obtained thanks to the use of ILROs as phase shifters.

#### 4.1.2. Effect of Process Variation

Process variation and mismatch may increase the temperature dependence of the sensor interface. 

**Figure 15:**
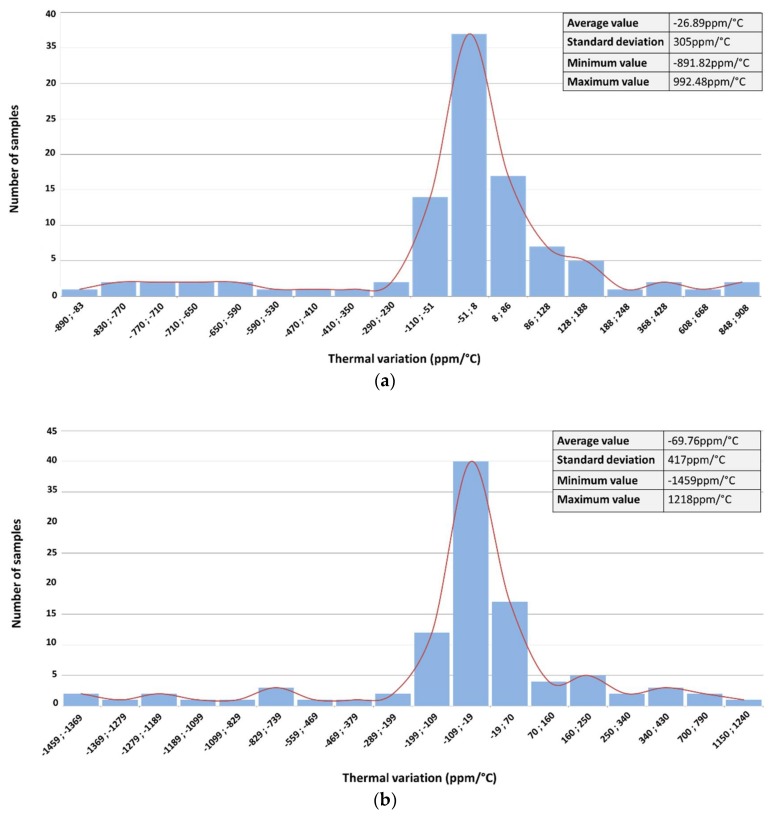
Monte Carlo simulation of the thermal stability of the sensor interface: (**a**) *V_S_* = 20 mV; (**b**) *V_S_* = 40 mV.

Thermal stability of the sensor interface over process and mismatch has been assessed with the help of Monte Carlo simulations. The obtained results are presented in Figure 15. For a *V_S_* of 20 mV, the average thermal variation over process and mismatch is close to that obtained in the simulation, while for a *V_S_* of 40 mV, the average thermal variation over process and mismatch is much higher than that obtained in typical simulation. It can be concluded that for a higher sensor output voltage *V_S_*, the effect of process variation and mismatch is more pronounced.

### 4.2. Characterizations of the Fabricated Prototype

**Figure 16:**
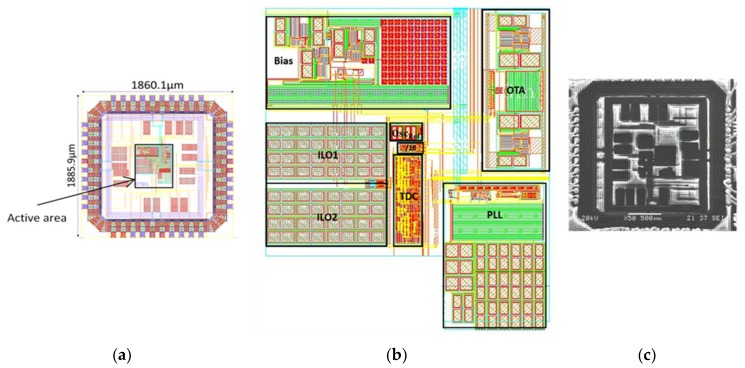
Sensor interface layout (**a**) Layout with the I/O rings; (**b**) Active area layout; (**c**) microphotography.

The wide-temperature-range sensor interface has been fabricated using the 0.18 μm Partially-Depleted Silicon on Insulator (PD-SOI) technology from XFAB France (Corbeil-Essonnes, France), (Figure 16).

**Figure 17:**
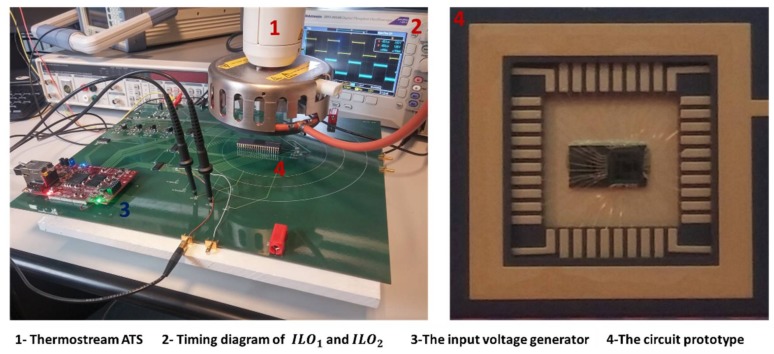
Characterization of the fabricated prototype.

For experimental measurements, the circuit has been packaged in a DIL-40 package and it has been tested on a high temperature printed circuit board (PCB). The sensor output voltage is emulated through an on-board differential signal generator (with no temperature dependence) and the prototype is placed under the nozzle of a thermal conditioner (Thermostream ATS, from Temptronic, Mansfield, MA, USA) to vary its temperature from −20 °C to 220 °C, which are the minimum and maximum temperatures of the thermal conditioner (Figure 17).

#### 4.2.1. Characteristic Function and Thermal Variation

**Figure 18:**
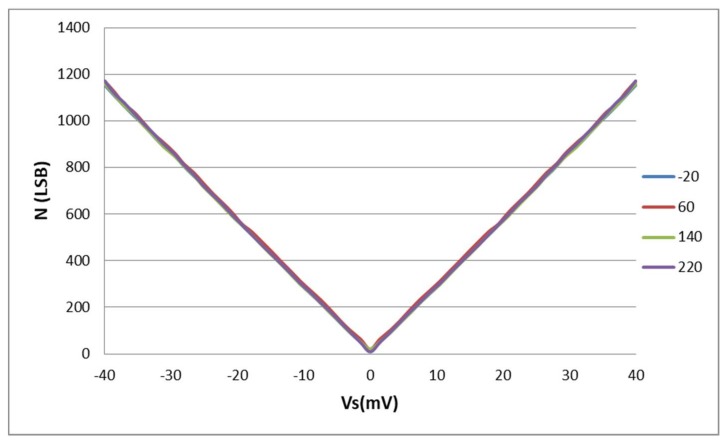
Experimental characteristic function of the sensor interface [−20 °C, 220 °C].

Figure 18 reports the digital output of the sensor interface as a function of the input voltage *V_S_* for different temperatures from −20 °C to 220 °C.

It is worth mentioning that no calibration circuit is used either to compensate the thermal variation of the sensor interface output or to calibrate the gain error and offset. Figure 18 shows the counter digital output that is proportional to the absolute value of the sensor output voltage *V_S_*. Nevertheless, the sign of *V_S_* is given by the available lead/lag bit (i.e., lead/lag between the two ILOs output signals) that permits to discriminate negative values of *V_S_* from positive by detecting which ILO output is in phase advance. This lead/lag bit, not shown in Figure 18, is at 1 logic level for a positive input voltage *V_S_* and at 0 logic level for a negative one.

**Figure 19:**
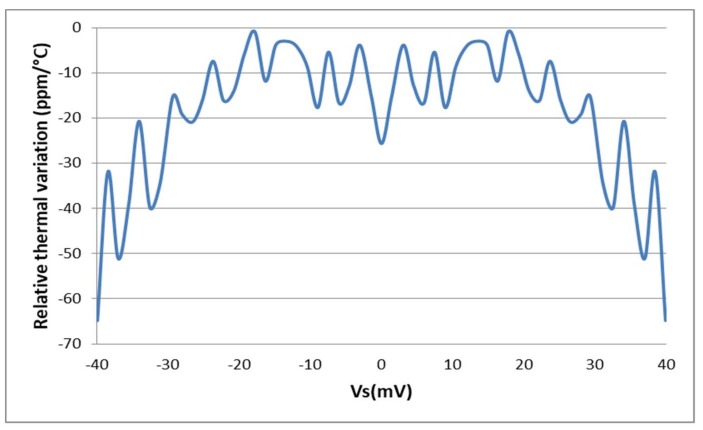
Measured relative thermal variation of the sensor interface.4.2. Linearity.

From the measured characteristic function of the fabricated prototype presented on Figure 19, the relative thermal variation is extracted using Equation (24). It shows that the circuit exhibits a low sensitivity to temperature variations; the absolute value of the measured relative thermal variation is always below 65 ppm/°C over the full operation temperature range (Figure 19), a value consistent with Monte Carlo simulation results of Figure 15.

Unlike what is obtained by simulations, the sensor interface output at zero *V_S_* has a non-zero thermal variation. This is because the fabricated sensor interface exhibits a certain output offset which is mainly due to mismatches and which is sensitive to temperature variations.

Precisely, the obtained offset is due to the mismatches inside the OTA which create a dissymmetry between its output currents. These mismatches could be reduced by using transistors with larger size.

#### 4.2.2. Linearity

**Figure 20:**
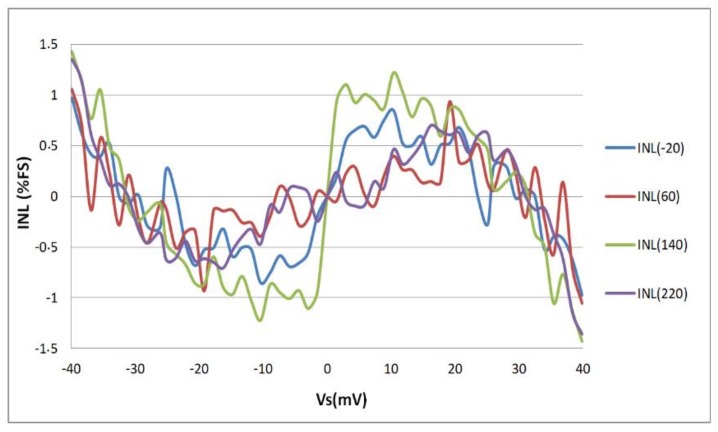
Measured integral non-linearity (INL) over a ±40 mV input dynamic range.

Over this wide operation temperature range, the fabricated sensor interface converts the differential input voltage *V_S_* into a digital output *N* with goodness-of-fit of linear regression higher than 0.998 (*R*^2^). Figure 20 depicts the difference between the measured values and the linear fits for different temperatures which represents the integral non-linearity of the circuit (INL). The maximum measured INL is lower than 1.5%. This value is obtained at the extremities of the input dynamic range. Figure 20 also shows that the measured INL is higher than the INL obtained in simulations (Figure 14). This may be caused by process variations.

As a conclusion, the performances of the fabricated sensor interface are similar to that obtained in both typical and Monte Carlo simulations (Figure 13 and Figure 15, respectively). The measured relative thermal variation is slightly higher than the relative thermal variation obtained by simulation (i.e., typical simulations presented in Figure 13). Nevertheless, the so-obtained relative thermal variation of the fabricated prototype is in good agreement with the Monte Carlo simulations results and the fabricated sensor interface achieves a low temperature dependency over a wide operation range.

The fabricated sensor interface does not however achieve the expected linearity; the measured INL is higher than simulated one. This may be explained by the effect of process variations. On one hand, process variations could degrade the linearity of the ILO transfer function *f*_0_(*I*_0_) (where *f*_0_ is its free running oscillation frequency and *I*_0_ is its bias current). On the other hand, the OTA linearity may be degraded by process variations. Since it is the first block of the sensor interface, this degradation could be at the origin of the high measured INL of the sensor interface. Moreover, the sensor interface has an open loop architecture. Then, a closed loop calibration could be considered in order to improve the linearity of the sensor interface. In order to better understand the origin of the high measured INL, further investigations are required.

However, it is not possible to bypass the use of OTA because the sensor output voltage conversion into a current is required in order to use current controled oscillators (CCOs) (i.e., ILROs) and to benefit from their high linear behaviour. The use of voltage controlled oscillators (VCOs) instead of CCOs could allow one to overcome the OTA imperfections. However, VCOs generally suffer from a reduced linearity compared to CCOs and this would degrade the INL of the sensor interface.

Table 1 summarizes the performances of the wide-temperature-range sensor interface compared to previous similar works. It shows that the presented sensor interface achieves good performances.

## 5. Conclusions

A time-domain differential sensor interface with a very large range of operating temperatures has been proposed. The concept is based on a fully differential architecture of the analog chain that leads to a very high immunity to temperature variations. Indeed, the digital output of the circuit depends only on the relative variation of few parameters and then only mismatches between identical components may induce some temperature dependence. 

A proof-of-concept has been designed and fabricated using a 0.18 µm partially-depleted SOI technology with 1.8 V power supply. Experimental results show that the sensor interface has an excellent temperature stability over a wide temperature range extending from −20 °C to 220 °C. The obtained result (−65 ppm/°C over an input range of ±40 mV) is, to our knowledge, the lowest temperature coefficient ever reported for a bit more than 11-bit resolution. 

An analysis based on experimental and Monte Carlo simulation results showed that the residual temperature dependence may be explained by mismatches. The use of larger area devices should permit to reduce this effect and thus to improve the level of performance.

## Figures and Tables

**Table 1:** Performances of the sensor interface.

Performances	This work	V. De Smedt, 2013 [2]	G. Glaser, 2017 [3]	V. De Smedt, 2012 [25]	Portmann, 2002 [26]	R. Grezaud, 2017 [27]
Temperature Range (°C)	−20 to 220 (Meas.) −40 to 250 (Sim.)	−20 to 100 (Meas.)	0 to 300 (Meas.)	−40 to 120 (Sim.)	25 to 300 (Meas.)	−40 to 180 (Meas.)
Thermal Drift	65 ppm/°C (Meas.) 38 ppm/°C (Sim.)	79 ppm/°C	±1.3%FS (±43 ppm/°C)	N.A	±4% of FS (±123 ppm/°C)	N.A
Sensor Type	resistive	resistive	Resistive	resistive	Magnetic	resistive
Non-Linearity	1.5% (Meas.)	0.7%	N.A	0.19%	N.A	N.A
Consumption	1 mW	18 μA	N.A	96 µW	4.5 mA	34 µA
Resolution	11 bit for the output +1 bit for the *V_S_* sign	N.A	N.A	9 bit	8 bit	10 bit
Size	1860.1 × 1885.9 (µm^2^) 0.21 mm^2^ (active)	550 × 300 (µm^2^) 95 × 95 (µm^2^) (active)	N.A	N.A	3.3 × 1.7 (mm^2^)	4.25 × 4.25 (mm^2^)
Technology	180 nm HT SOI Vdd ^1^: 1.8 V	40 nm CMOS Vdd: 1 V	N.A	130 nm CMOS Vdd: 1.2 V	1 µm CMOS Vdd: 5 V	180 nm HT SOI Vdd: 1.8 V

^1^ Vdd is the supply voltage of the technology.
